# Supplementary material for: EEG theta and alpha oscillations during tactical decision-making: An examination of the neural efficiency hypothesis in volleyball
Source: PLoS One. 2025 Feb 13;20(2):e0318234. doi: 10.1371/journal.pone.0318234 (PMC11825103; doi:10.1371/journal.pone.0318234)
Supplement: S1 Tables — Additional tables showing complementary information and analyses. (PDF) [file pone.0318234.s001.pdf]

**Table A. Deviations from preregistration.**

| Method: | Prereg: | Article: |
|---|---|---|
| Sample Size | "For this study we plan to recruit 25 participants for each of the three groups "experts", "amateurs" and "novices", which will result in a total of 75 participants. The sex ratio will be counterbalanced within each group. In their review on self-paced sports Filho et al. (2021) find that the effect sizes for group differences in ERD/S of novices and experts, are located around g = 0.89 for Alpha (experts > novices) and g = 0.91 for Theta (experts < novices). For a significant group difference with a significance level of 0.05 (two-tailed) and power of 0.80 this results in a predicted minimum sample size of 20-21 participants per group, as calculated with G*Power (Ver. 3.1.9.6; Faul et al., 2007)." | As recruitment of professional athletes is a difficult endeavor, we were only able to achieve a group size of 16 experts. Together with the 22 novices and 26 amateurs, our sample in total included 64 participants. Although a higher number of experts would have been preferable, our sample of experts is comparable in size to those of previous and even more recent studies in the literature (Babiloni et al., 2009; DeCouto et al., 2023; Del Percio et al., 2019). This makes our results comparable to these previous findings, which is why we deem our results valid for interpretation. Furthermore, since we were unable to find reliable sources for effect sizes from a three-expertise-group design for EEG Alpha or Theta, we used the effect sizes determined in the review by Filho et al. (2021) as a reference for our power calculations, which are however based on a two-expertise-group design. Considering that these effect sizes are rather large (Alpha: g = 0.89; Theta: g = 0.91), when converting them to Cohen's f and calculating the estimated power for a between factor effect in our factorial design (3 groups, 4 measurments points per participant) and with a total group size of 48 (16 per group), we would still achieve a high statistical power of 0.93 to find a significant between factor effect, as calculated with G*Power (Ver. 3.1.9.7; Faul et al., 2007). |

| | | |
|---|---|---|
| Analyses | "For the evaluation of between- ("expertise") and within-subjects ("condition", "perspective") effects as well as interaction and random effects (subjects), behavioral and neurophysiological data will be analyzed by means of linear mixed-effect models. " | As we encountered difficulties with the definition of linear mixed-effect models with our factorial design, we chose to revert to a simpler general linear model design. Furthermore, factor names were changed as follows: "expertise" >> "Group", "condition" >> "Task", "perspective" >> "Court"; |
| Outliers and Exclusions | "The following EEG exclusion criteria will be set: (i) > 50 µV voltage difference between two data points, (ii) > 200 µV voltage difference within a 200 ms interval, and (iii) absolute voltage values ± 120 µV." | To have less data excluded from automatic artifact identification, a value of ± 200 µV was used for criterion (iii) of the EEG preprocessing pipeline. |

Methods that were preregistered for this study at the Open Science Framework (https://osf.io/exhr3), for which the actual implementation in the article deviated are described under "Prereg". Implemented procedures are described under "Article".

References:


Babiloni, C., Del Percio, C., Rossini, P. M., Marzano, N., Iacoboni, M., Infarinato, F., Lizio, R., Piazza, M., Pirritano, M., Berlutti, G., Cibelli, G., & Eusebi, F. (2009). Judgment of actions in experts: A high-resolution EEG study in elite athletes. *NeuroImage*, *45*(2), 512–521. https://doi.org/10.1016/j.neuroimage.2008.11.035

DeCouto, B. S., Smeeton, N. J., & Williams, A. M. (2023). Skilled Performers Show Right Parietal Lateralization during Anticipation of Volleyball Attacks. *Brain Sciences*, *13*(8), 1204. https://doi.org/10.3390/brainsci13081204

Del Percio, C., Franzetti, M., De Matti, A. J., Noce, G., Lizio, R., Lopez, S., Soricelli, A., Ferri, R., Pascarelli, M. T., Rizzo, M., Triggiani, A. I., Stocchi, F., Limatola, C., & Babiloni, C. (2019). Football Players Do Not Show "Neural Efficiency" in Cortical Activity Related to Visuospatial Information Processing During Football Scenes: An EEG Mapping Study. *Frontiers in Psychology*, *10*(APR). https://doi.org/10.3389/fpsyg.2019.00890

Faul, F., Erdfelder, E., Lang, A. G., & Buchner, A. (2007). G*Power 3: A flexible statistical power analysis program for the social, behavioral, and biomedical sciences. *Behavior Research Methods*, *39*(2), 175–191. https://doi.org/10.3758/BF03193146

Filho, E., Dobersek, U., & Husselman, T.-A. (2021). The role of neural efficiency, transient hypofrontality and neural proficiency in optimal performance in self-paced sports: a meta-analytic review. *Experimental Brain Research*, *239*(5), 1381–1393. https://doi.org/10.1007/s00221-021-06078-9

**Table B. Descriptions of questionnaire variables.**

| Name: | Name in CSV: | Description: |
|---|---|---|
| SOC01 | code | ID code of the participant |
| SOC02 | group | Expertise group category (Novice/Amateur/Expert) |
| SOC03 | age | Age in years |
| SOC04 | sex | Sex (female/male) |
| SOC05 | handedness | Self-reported handedness (left/right) |
| SOC06 | vb_active | “Do you currently play actively in a volleyball club?” (no/yes) |
| SOC07 | vb_team | “Have you ever played in a volleyball club?” (no/yes) |
| SOC08 | vb_leisure | “Do you play volleyball in your free time?” (no/yes) |
| SOC09 | vb_usi_course | “Are you currently attending one or more volleyball courses at the University Sports Institute (USI)?” (no/yes) |
| SOC10 | vb_usi_level | “What level are you on? (If you attend several volleyball USI courses, please indicate the highest level.)” (beginner/intermediate/advanced) |
| SOC11 | vb_times_week | “On average, how many times a week do you play volleyball?” (0 to 1 times/2 to 3 times/4 to 5 times/more than 5 times) |
| SOC12 | vb_h_week | “On average, how many hours a week do you play volleyball? (Please provide an approximate estimate of the number of hours.)” |
| SOC13 | vb_years | “Approximately how many years of volleyball experience do you have? (Please provide a rough estimate of the years.)” |
| SOC14 | vb_rules | “How much knowledge do you have on the rules of volleyball?” (none/little/some/much) |
| SOC15 | vb_watch | “How often have you attended volleyball tournaments as a spectator or watched them on TV in the last 10 years?” (0 to 1 times/2 to 3 times/4 to 5 times/more than 5 times) |
| SOC16 | sport | “Apart from volleyball, do you play sports regularly?” (no/yes) |
| SOC17 | sport_times_week | “On average, how many times a week do you exercise?” (0 to 1 times/2 to 3 times/4 to 5 times/more than 5 times) |

| | | |
|---|---|---|
| SOC18 | sport_h_week | "On average, how many hours per week do you exercise? (Please provide an approximate estimate of the number of hours.)" |
| EHI | ehi_score | Laterality index from the Edinburgh Handedness Inventory (EHI; Oldfield, 1971): Values range from -100 (fully left-handed) to +100 (fully right-handed), meaning high negative values are associated with left-handedness, values around 0 with ambidexterity and high positive values with right-handedness. |
| FLOW_S | flow_short_scale | Flow Short Scale: Measure of the extent to which a person felt the experience of flow during a previously completed task. Values range from 1 (low flow) to 7 (high flow). |
| FLOW_C | flow_concern_scale | Flow Concern Scale: Measure of the extent to which a person had concerns regarding their performance during a previously completed task. Values range from 1 (low concern) to 7 (high concern). |
| VAS01 | vas_01 | "How difficult did you find it to predict the pass position when the play was happening on the closer half of the court?" (Not difficult at all/Very difficult) |
| VAS02 | vas_02 | "How difficult did you find it to identify the position of the service player when the play was happening on the closer half of the court?" (Not difficult at all/Very difficult) |
| VAS03 | vas_03 | "How difficult did you find it to predict the pass position when the play was happening on the farther half of the court?" (Not difficult at all/Very difficult) |
| VAS04 | vas_04 | "How difficult did you find it to identify the position of the service player when the play was happening on the farther half of the court?" (Not difficult at all/Very difficult) |
| VAS05 | vas_05 | "How motivated were you to complete the tasks?" (Not motivated at all/Very motivated) |
| VAS06 | vas_06 | "How did your motivation change over the course of the testing when working on the tasks?" (Decreased significantly/Increased significantly) |
| VAS07 | vas_07 | "How concentrated were you during the tasks?" (Not at all concentrated/Very concentrated) |
| VAS08 | vas_08 | "How has your concentration changed over the course of the testing when working on the tasks?" (Decreased significantly/Increased significantly) |

| | | |
|---|---|---|
| VAS09 | vas_09 | "While watching the game scenes, were you able to identify patterns that helped you with the tasks?" (Not at all/Very much) |
| VAS10 | vas_10 | "How much do you think your previous knowledge of volleyball helped you with the tasks?" (Not at all/Very much) |
| VAS11 | vas_11 | "When predicting the outcome of the game scenes, did you feel more like guessing or knowing the answer?" (Guess/Know) |
| VAS12 | vas_12 | "Did you feel any pain during the testing?" (Not at all/Very bad) |

Information refers to the data file "SOC_FLOW_VAS_Data.csv" available in the online materials of this article (https://osf.io/ck5zu/). Given answer options for categorical variables are presented at the end of the description in parentheses. In the case of visual analogue scale (VAS) items, the labels for the respective low (0) and high (100) ends are presented in parentheses.

**Table C. Visual analog scale (VAS) analysis results.**

| | Group: | | | Statistics: | | | |
|---|---|---|---|---|---|---|---|
| Variable: | Novice ($n$ = 22) | Amateur ($n$ = 26) | Expert ($n$ = 16) | $df$ | $F$ | $p$ | $\eta_p^2$ |
| "How difficult did you find it to predict the pass position when the play was happening on the closer half of the court?" (Not difficult at all/Very difficult) [VAS01] ** | 46.2 (±27.8) | 53.0[a] (±21.0) | 30.0[a] (±17.4) | 2, 61 | 5.08 | .009 | .143 |
| "How difficult did you find it to identify the position of the service player when the play was happening on the closer half of the court?" (Not difficult at all/Very difficult) [VAS02] | 6.7 (±7.1) | 9.0 (±11.9) | 3.0 (±3.5) | 2, 61 | 2.24 | .115 | .069 |
| "How difficult did you find it to predict the pass position when the play was happening on the farther half of the court?" (Not difficult at all/Very difficult) [VAS03] ** | 57.8[a] (±26.2) | 56.7[b] (±21.2) | 32.7[ab] (±16.4) | 2, 61 | 7.44 | .001 | .196 |
| "How difficult did you find it to identify the position of the service player when the play was happening on the farther half of the court?" (Not difficult at all/Very difficult) [VAS04] | 12.5 (±18.4) | 12.0 (±12.6) | 4.4 (±5.1) | 2, 61 | 1.94 | .152 | .060 |
| "How motivated were you to complete the tasks?" (Not motivated at all/Very motivated) [VAS05] | 76.2 (±16.6) | 78.2 (±18.9) | 84.8 (±13.1) | 2, 61 | 1.27 | .289 | .040 |
| "How did your motivation change over the course of the testing when working on the tasks?" (Decreased significantly/Increased significantly) [VAS06] | 42.2 (±23.5) | 41.3 (±16.6) | 47.5 (±16.5) | 2, 61 | 0.56 | .575 | .018 |
| "How concentrated were you during the tasks?" (Not at all concentrated/Very concentrated) [VAS07] | 68.4 (±20.5) | 74.0 (±16.5) | 78.0 (±16.3) | 2, 61 | 1.38 | .260 | .043 |

| | | | | | | | |
|---|---|---|---|---|---|---|---|
| "How has your concentration changed over the course of the testing when working on the tasks?" (Decreased significantly/Increased significantly) [VAS08] | 36.2 (±21.5) | 39.0 (±18.0) | 39.0 (±12.1) | 2, 61 | 0.18 | .837 | .006 |
| "While watching the game scenes, were you able to identify patterns that helped you with the tasks?" (Not at all/Very much) [VAS09] | 57.5 (±27.5) | 62.7 (±29.0) | 75.2 (±25.2) | 2, 61 | 1.98 | .147 | .061 |
| "How much do you think your previous knowledge of volleyball helped you with the tasks?" (Not at all/Very much) [VAS10] *** | $46.5^{ab}$ (±35.7) | $69.4^{ac}$ (±26.6) | $84.7^{bc}$ (±16.3) | 2, 61 | 8.93 | < .001 | .226 |
| "When predicting the outcome of the game scenes, did you feel more like guessing or knowing the answer?" (Guess/Know) [VAS11] ** | $55.2^{a}$ (±25.6) | $52.2^{b}$ (±20.9) | $73.3^{ab}$ (±16.5) | 2, 61 | 5.09 | .009 | .143 |
| "Did you feel any pain during the testing?" (Not at all/Very bad) [VAS12] | 9.0 (±17.7) | 8.6 (±17.1) | 1.8 (±2.1) | 2, 61 | 1.30 | .279 | .041 |

Visual analogue scale (VAS) item labels are presented in the "Variable" column; labels for the respective low (0) and high (100) ends are presented in parentheses. Variable names are provided in subscript square brackets. Group means are presented for each VAS variable, with standard deviations in parentheses in the "Group" column. ANOVA results are presented in the "Statistics" column. Variables with significant overall ANOVA results are indicated with asterisks (**: $p < .01$; ***: $p < .001$). Significant group differences, as determined through pairwise *t*-tests, are indicated with uppercase letters ($^{a\text{-}c}$).

**Table D. Gravity individual alpha frequency (IAF) analysis results.**

| | Group: | | | Statistics: | | | |
|---|---|---|---|---|---|---|---|
| **Variable:** | Novice | Amateur | Expert | *df* | *F* | *p* | $\eta_p^2$ |
| Eyes-open | 9.9 (±0.5) | 9.8 (±0.2) | 9.8 (±0.5) | 2, 57 | 0.37 | .695 | .013 |
| Eyes-closed | 9.9 (±0.5) | 10.1 (±0.5) | 9.8 (±0.4) | 2, 56 | 1.44 | .245 | .049 |

Group means are presented for Eyes-open and Eyes-closed gravity IAF calculations, with standard deviations in parentheses in the "Group" column. ANOVA results are presented in the "Statistics" column.
