# Supplementary material for: EEG theta and alpha oscillations during tactical decision-making: An examination of the neural efficiency hypothesis in volleyball
Source: PLoS One. 2025 Feb 13;20(2):e0318234. doi: 10.1371/journal.pone.0318234 (PMC11825103; doi:10.1371/journal.pone.0318234)
Supplement: Attachment — Submitted filename: Review- PONE-D-24-33452.docx [file pone.0318234.s002.docx]

Manuscript title: EEG theta and alpha oscillations during tactical decision-making: An examination of the neural efficiency hypothesis in volleyball

**Recommendation (Answer options: Accept, Minor Revision, Major Revision, Reject):** Minor Revision **2. Is the manuscript technically sound, and do the data support the conclusions? (Answer options: Yes, No, Partly).** yes **3. Has the statistical analysis been performed appropriately and rigorously? (Answer options: Yes, No, I don’t know, N/A).** yes **4. Does the manuscript adhere to the PLOS Data Policy? Additional details can be found at http://journals.plos.org/plosone/s/materials-and-software-sharing. (Answer options: Yes, No).** yes **5. Is the manuscript presented in an intelligible fashion and written in standard English? (Answer options: Yes, No).** I am not native but I found the manuscript difficult to understand (especially the Introduction and Discussion Sections). The writing style could be improved by using clearer language**.
6. Review Comments to the Author (minimum 200 characters):**

This study (EEG theta and alpha oscillations during tactical decision-making: An examination of the neural efficiency hypothesis in volleyball) examined how expertise in volleyball affects brain activity during sport-related decision-making. Participants with different levels of experience (three groups) were asked to predict the outcome of volleyball plays. Researchers measured theta and alpha waves, to assess neural efficiency. The results showed that more experienced players were better at making accurate predictions. Both experienced and less experienced players showed increased brain activity during the task, but there were no significant differences between groups. While these findings suggest that expertise is linked to improved performance, the relationship between expertise and brain efficiency in this specific context is more nuanced than the neural efficiency hypothesis might predict.

**Introduction:**

The research question is clear and relevant. The authors have effectively outlined the theoretical framework, particularly the neural efficiency hypothesis, and its application to the domain of sport. Minor comment is that in Page 5, line 76, when starting to argument the relationship between theta and alpha power changes and their association with task performance, it would be beneficial to provide a clearer and more detailed explanation, especially for readers who may not be experts in the EEG field.

**Method:**

The researchers have employed a rigorous and appropriate research design to address the research question. The statistical analyses are robust and the data analysis is well-conducted.

**Results:**

The results are clearly presented. Minor comment: to further strengthen the interpretation of the results, it would be beneficial to visualize the time-course of theta and alpha power changes using Time-Frequency Representations. This would provide a more detailed understanding of the dynamic changes in brain activity during the task.

**Discussion:**

The discussion is well-argued but the writing style could be improved by using clearer language and avoiding overly complex terminology.

**7. PLOS authors have the option to publish the peer review history of their article (https://journals.plos.org/plosone/s/editorial-and-peer-review-process#loc-peer-review-history). If published, this will include your full peer review and any attached files. Do you want your identity to be public for this peer review? (Answer options: Yes, No).** yes **7a. If you answered "Yes" above, that you would like your identity to be public, please provide your full name, as it should appear on the published peer review. Please do not sign the review on behalf of another person.** Mohammad Ali Nazari **Confidential to Editor:
1. Do you have any potentially competing interests? If none, type "None." Our policy on competing interests can be found at** [**http://journals.plos.org/plosone/s/competing-interests**](http://journals.plos.org/plosone/s/competing-interests)**.** None **2. Did you receive any assistance in preparing this review (e.g. from a post-doc or graduate student)? If yes, please provide their name and title. If you have chosen to reveal your identity to the authors in question 7 above, please include the name of the person who assisted you in the response to question 7 as well.**  No **3. Do you want to receive recognition for this review on a Web of Science researcher profile? (https://clarivate.com/products/scientific-and-academic-research/research-discovery-and-workflow-solutions/researcher-profiles/)? If you opt in, your Web of Science profile will automatically be updated to show a verified record of this review in full compliance with the journal's review policy. If you don't have a Web of Science profile, you will need to first create a free account. (Answer options: Yes, No).** yes
